# Editorial: Multi-modality imaging and multi-omics approach to pediatric neurogenetic disorders

**DOI:** 10.3389/fninf.2023.1192211

**Published:** 2023-05-03

**Authors:** Xinran Dong

**Affiliations:** Center for Molecular Medicine, Children's Hospital of Fudan University, Shanghai, China

**Keywords:** pediatric genetic disorder, multi-modalities imaging, multi-omics, data integration, disease biomarker

Pediatric neurogenetic disorders encompass a wide range of conditions caused by genetic or chromosomal changes, impacting the brain, spinal cord, nerves, and muscles. These disorders can lead to various symptoms, such as intellectual disability, developmental delay, seizures, movement disorders, and other neurological and behavioral issues. Children with neurogenetic disorders often undergo complex diagnostic journeys. As a result, optimal diagnosis and management necessitate a multidisciplinary team.

Two emerging fields in medical research, multi-modality imaging and multi-omics approaches, aim to enhance the diagnosis, treatment, and understanding of these disorders. Multi-modality imaging combines multiple imaging techniques like MRI, CT, PET, and SPECT to provide a comprehensive view of the brain and nervous system's structure and function. Meanwhile, multi-omics approaches integrate different types of omics data, including genomics, transcriptomics, epigenomics, proteomics, and metabolomics, to better understand the molecular mechanisms behind pediatric neurogenetic disorders.

This Research Topic comprises four articles: three focus on multi-modality imaging, while one employs a multi-omics approach.

The first article is titled “*Brain structural alterations in young girls with Rett syndrome: A voxel-based morphometry and tract-based spatial statistics study*” (Li et al.). Rett syndrome (RTT) is a neurodevelopmental disorder caused by loss-of-function variants in *MECP2* gene, currently with no cure. The purpose of the study was to explore brain structural changes in girls with RTT. The authors used voxel-based morphometry (VBM) and tract-based spatial statistics (TBSS) methods to compare gray and white matter differences in-between girls with Rett syndrome and age- and gender-matched girls with autism and typical development. Results showed that, in RTT, decreased gray matter volume were found in insula, frontal cortex, calcarine and limbic/paralimbic regions; and altered white matter parameters were mainly located in corpus callosum, superior longitudinal fasciculus, corona radiata, and etc. This monogenic study with early stage of RTT provides valuable guidance for understanding the disease pathogenesis. At the same time, the pediatric-adjusted analytic pipelines for VBM and TBSS were introduced for significant improvement over classical approaches for MRI scans in children.

The second article “*Alteration of brain nuclei in obese children with and without Prader-Willi syndrome*” aims to investigate the differences in brain nuclei between obese children with and without Prader-Willi syndrome (PWS) (Wu et al.). PWS is a genetic disorder caused by loss of paternal gene expression located in chromosome 15q11-q13. The purpose of this study is to testify the hypothesis that Children with PWS would exhibit genetically influenced impairment in brain nuclei compared with the controls whereas no significant difference was found for the obesity group. The authors used T1-weighted magnetic resonance imaging to investigate alterations in brain nuclei by three automated analysis methods and found that children with PWS had significant atrophy in several brain regions, whereas no significant difference was found between the other two groups. Specially, the dysfunction of deep cerebellar nuclei (DCN) contributed to the developmental behavioral characteristics in PWS. The study highlights the potential mechanism associated with clinical manifestations of PWS, distinct from the neural mechanisms of obese children.

The article “*Temporal and spatial dynamic propagation of electroencephalogram by combining power spectral and synchronization in childhood absence epilepsy*” aims to investigate the dynamic propagation of electroencephalogram (EEG) signals in children with absence epilepsy (Zhong et al.). Childhood Absence epilepsy (CAE) is a type of epilepsy that causes brief lapses in consciousness, which accounts for 10–17% of all pediatric epilepsy. EEG recordings is an essential tool for clinical diagnosis and treatment. The purpose of the study is to find quantitative EEG markers to distinguish among normal, pre-ictal, and ictal states, which can be used for the detection and early warning of CAE seizures. The authors confirmed that power, frequency, and spatial synchronization are enhanced during CAE and rapid diffusion occurs in the frontal region, with EEG power rapidly bursting throughout the brain within a few seconds after the onset. The study concludes that CAE seizures are related to abnormal discharge diffusion and synchronization network, and the temporal and spatial evolution of EEG is essential for clinical diagnosis and automatic detection of CAE.

The article “*Multi-omics integration reveals a six-malignant cell maker gene signature for predicting prognosis in high-risk neuroblastoma*” aims to identify a gene signature that can predict the prognosis of high-risk neuroblastoma, a type of cancer that mainly affects young children (Yan et al.). The study used multi-omics analysis, which combines transcriptomic, and epigenomic data, to identify genes that are consistently dysregulated in high-risk neuroblastoma. Using this approach, the authors identified six genes that were consistently upregulated in high-risk neuroblastoma samples compared to low-risk samples, which can be a strong predictor of prognosis, with patients with high expression levels having significantly worse outcomes. Overall, the study provides insight into the molecular mechanisms underlying high-risk neuroblastoma and highlights the potential of using multi-omics analysis to identify gene signatures that can predict prognosis and guide treatment decisions.

The use of multi-modality imaging and multi-omics approaches in the study of pediatric neurogenetic disorders represents an exciting new frontier in medical research. By combining information from multiple imaging techniques and omics data, researchers can obtain a more comprehensive view of the structure, function, and molecular mechanisms underlying these disorders. However, there are also challenges associated with the use of these approaches in pediatric neurogenetic disorders. Additionally, there may be ethical and legal issues surrounding the use of these approaches in pediatric patients, particularly with regards to informed consent and data privacy. Despite these challenges, the potential benefits of using multi-modality imaging and multi-omics approaches in the study of pediatric neurogenetic disorders are significant. The discovery of disease biomarkers and exploration of disease mechanisms are crucial for advancing our understanding and treatment of pediatric neurological genetic diseases. The articles in this Research Topic offer valuable insights and exemplary approaches in this regard. We hope that their findings will inspire and encourage more researchers to pursue innovative and collaborative ways of integrating data and contributing to this field.

## Author contributions

The author confirms being the sole contributor of this work and has approved it for publication.

